# The Underrecognized Role of Cannabis in the Etiology of Acute Pancreatitis

**DOI:** 10.7759/cureus.68612

**Published:** 2024-09-04

**Authors:** FNU Tanvir, Sumerjit Singh, Kanwarmandeep Singh, Chidera N Onwuzo, Jaskaran Singh, Harman Antaal, Ajay Pal Singh Sandhu, Meet Sirjana Kaur, Harmanjot Singh, Agamjit Singh

**Affiliations:** 1 Internal Medicine, Government Medical College Amritsar, Amritsar, IND; 2 Diagnostic Radiology, Government Medical College Amritsar, Amritsar, IND; 3 Medicine, Government Medical College Amritsar, Amritsar, IND; 4 Internal Medicine, State University of New York Upstate Medical University, Syracuse, USA; 5 Internal Medicine, Benjamin S. Carson College of Health and Medical Sciences, Ilishan-Remo, NGA; 6 Internal Medicine, Lagos Island General Hospital, Lagos, NGA; 7 Internal Medicine, Sri Guru Ram Das University of Health Sciences, Amritsar, IND; 8 Internal Medicine, Government Medical College, Patiala, Patiala, IND; 9 Internal Medicine, The White Medical College and Hospital, Pathankot, IND; 10 Psychiatry, Punjab Institute of Medical Sciences, Jalandhar, IND

**Keywords:** pancreatic inflammation, cannabis cessation, chronic pancreatitis, recurrence, management, diagnosis, cannabinoid receptors, etiology, acute pancreatitis, cannabis

## Abstract

Cannabis-induced pancreatitis (CIP) is an emerging clinical entity that presents unique challenges in diagnosis and management. This narrative review explores the current understanding of CIP, synthesizing evidence from epidemiological, pathophysiological, and clinical studies. The rising prevalence of cannabis use worldwide has been paralleled by an increase in reported cases of CIP, particularly among younger populations. Pathophysiological mechanisms involve the interaction of exogenous cannabinoids with pancreatic cannabinoid receptors, potentially disrupting normal pancreatic function and triggering inflammation. Clinical presentation of CIP often mimics other forms of acute pancreatitis (AP), necessitating a high index of suspicion and thorough history-taking for accurate diagnosis. Management strategies align with established protocols for AP, with an emphasis on supportive care and cannabis cessation to prevent recurrence. While short-term outcomes are generally favorable, the risk of progression to chronic pancreatitis in cases of continued cannabis use underscores the importance of long-term follow-up and abstinence counseling. This review also highlights significant knowledge gaps, including the need for standardized diagnostic criteria, a better understanding of dose-response relationships, and potential interactions with other risk factors. Future research directions should focus on elucidating precise pathophysiological mechanisms, developing targeted therapies, and investigating the impact of different cannabis formulations and consumption methods on pancreatic health. As cannabis use continues to increase globally, a comprehensive understanding of its effects on pancreatic function is crucial for improving patient outcomes and informing public health policies.

## Introduction and background

Cannabis use has experienced a significant surge in popularity over the past few decades, with an estimated 209 million users worldwide as of 2020 [1]. This increasing prevalence can be attributed to various factors, including changing social attitudes, legalization efforts in several countries, and growing interest in its potential therapeutic applications [2]. As cannabis consumption becomes more widespread, it is crucial to investigate its impact on various physiological systems, including the gastrointestinal tract and associated organs.

One area of particular concern is the potential relationship between cannabis use and acute pancreatitis (AP). AP is a sudden inflammatory condition of the pancreas, characterized by severe abdominal pain, nausea, and vomiting [3]. While the most common etiologies of AP include gallstones and alcohol abuse, accounting for approximately 70-80% of cases, there remains a significant proportion of cases with unclear or idiopathic origins [4]. Recent evidence suggests that cannabis may play an underrecognized role in the etiology of AP, particularly in younger populations where traditional risk factors are less prevalent [5].

Pancreatitis, in its acute form, can range from mild and self-limiting to severe and life-threatening. The condition is associated with significant morbidity and mortality, with severe cases potentially leading to systemic inflammatory response syndrome, multi-organ failure, and death [6]. The global incidence of AP has been steadily increasing, with estimates ranging from 13 to 45 cases per 100,000 individuals annually [7]. This rising incidence, coupled with the substantial healthcare burden associated with managing AP, underscores the importance of identifying and understanding all potential risk factors, including the possible role of cannabis.

As research into the effects of cannabis on various organ systems continues to expand, it is becoming increasingly apparent that its impact on pancreatic function and inflammation warrants closer examination. By exploring the potential link between cannabis use and AP, we may uncover valuable insights that could inform clinical practice, guide patient education efforts, and contribute to more comprehensive strategies for preventing and managing this serious condition.

## Review

Epidemiology of cannabis-induced pancreatitis (CIP)

The epidemiology of CIP is an emerging field of study, with researchers increasingly recognizing the potential link between cannabis use and AP. While the exact prevalence and incidence of CIP remain challenging to determine due to underreporting and the often multifactorial nature of pancreatitis, several studies have shed light on this growing concern.

A retrospective analysis of hospital admissions in the United States revealed a significant increase in cannabis-related AP cases over the past decade [8]. The study found that the incidence of CIP rose from 0.3 cases per 100,000 admissions in 2006 to 2.8 cases per 100,000 admissions in 2015, representing a nearly 10-fold increase. This trend parallels the rising prevalence of cannabis use in the general population, suggesting a potential causal relationship [2].

Furthermore, a multicenter study conducted across five European countries reported that cannabis use was associated with 8% of idiopathic AP cases [9]. This finding underscores the importance of considering cannabis as a potential etiological factor in cases where traditional risk factors are absent.

**Figure 1:**
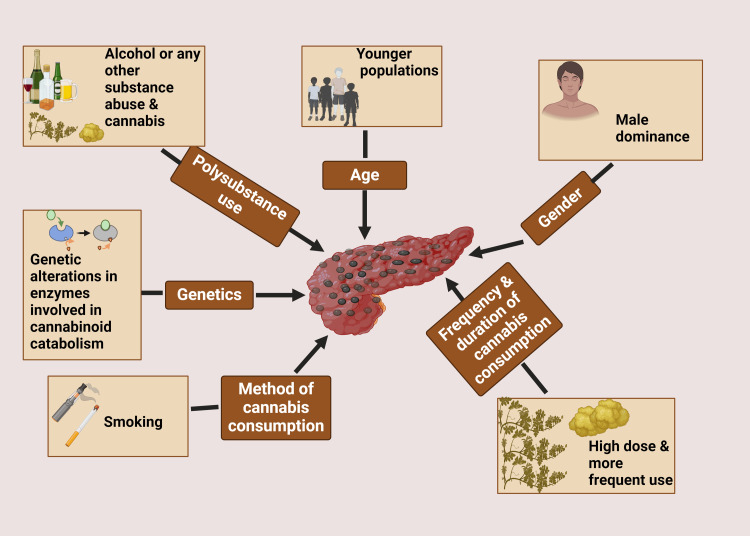
Risk factors associated with cannabis-induced pancreatitis Figure created in BioRender.com.

Several risk factors and demographic patterns have been identified in relation to CIP (Figure 1). Age appears to be a significant factor, with younger individuals being more susceptible. A systematic review of case reports and case series found that the median age of patients with cannabis-induced AP was 24.5 years [5]. This demographic pattern differs notably from the typical age distribution of AP caused by other etiologies, which tends to affect older populations.

Gender also plays a role in the epidemiology of CIP. Studies have consistently reported a male predominance, with men accounting for approximately 70-80% of cases [10]. This gender disparity may be partly explained by the higher prevalence of cannabis use among males compared to females in many populations.

The frequency and duration of cannabis use appear to be important risk factors. Chronic, heavy cannabis use is more commonly associated with AP than occasional or light use [5]. A dose-dependent relationship has been suggested, with higher doses and more frequent use correlating with an increased risk of pancreatic inflammation.

Interestingly, the method of cannabis consumption may also influence the risk of developing AP. Inhalation, particularly smoking, has been reported as the most common route of administration in cases of CIP [11]. This observation raises questions about whether the combustion products of cannabis smoking may contribute to pancreatic injury, or if the rapid absorption and high peak concentrations achieved through inhalation play a role in the pathogenesis.

Genetic factors may also contribute to individual susceptibility to CIP. Some researchers have proposed that genetic variations in cannabinoid receptors or in enzymes involved in cannabinoid metabolism could modulate the risk of developing pancreatitis in cannabis users [12]. However, more research is needed to elucidate the specific genetic markers and their clinical significance.

It is important to note that CIP often occurs in the context of polysubstance use. Many reported cases involve concurrent alcohol consumption or the use of other illicit substances, which may have synergistic effects on pancreatic inflammation [13]. This complicates the epidemiological picture and highlights the need for careful consideration of confounding factors in future studies.

As cannabis legalization continues to expand globally, it is crucial to monitor the epidemiological trends of CIP closely. Improved surveillance, standardized reporting, and prospective studies are needed to better quantify the true prevalence and incidence of this condition and to identify additional risk factors that may inform prevention strategies and clinical management.

Pathophysiology

**Figure 2:**
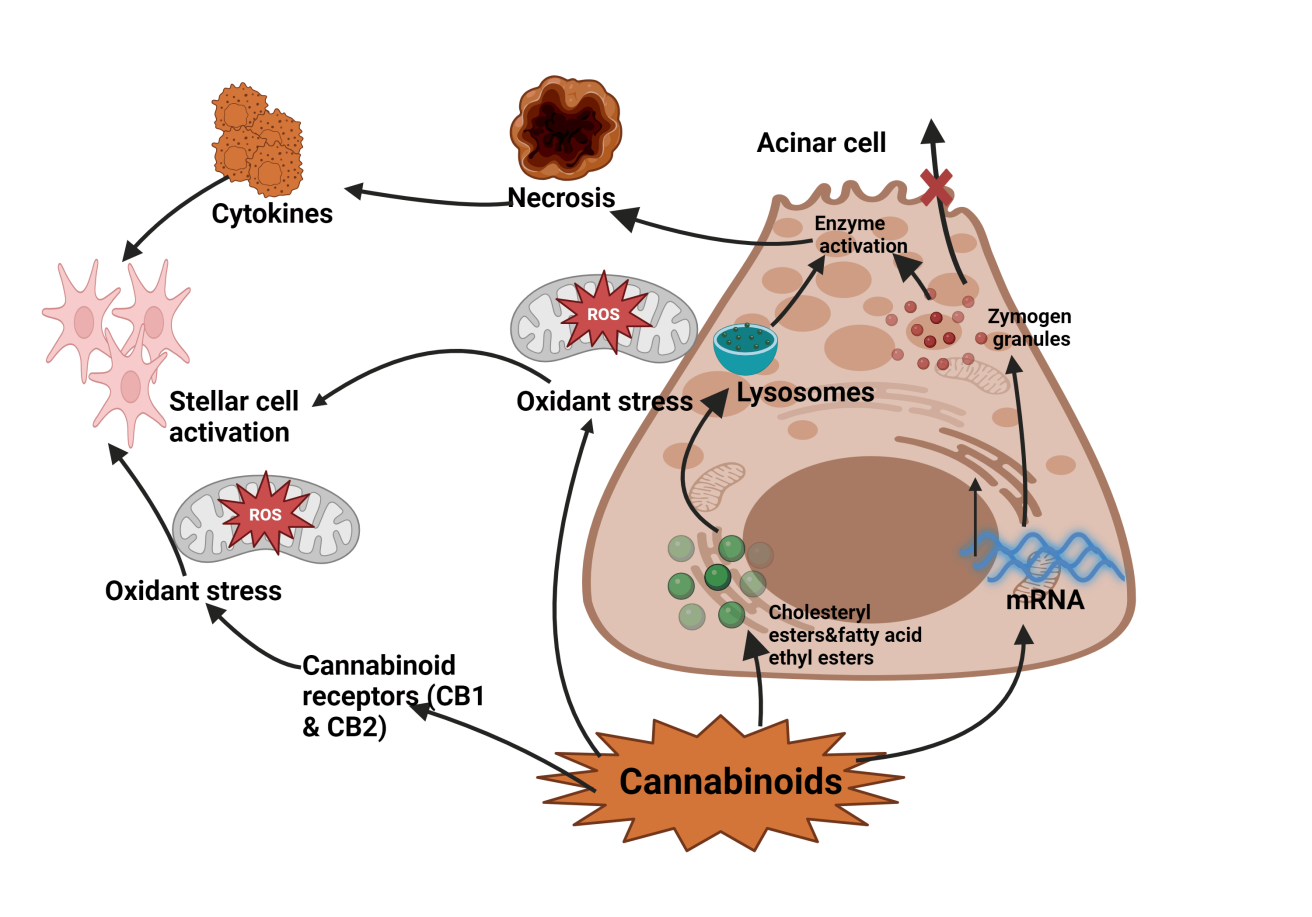
Pathophysiology of cannabis-induced pancreatitis The pathogenesis of cannabis-induced pancreatitis begins with cannabinoids affecting subcellular organelles, increasing digestive and lysosomal enzyme content while destabilizing lysosomes and zymogen granules. These changes sensitize cells to trigger factors, potentially leading to acute pancreatitis. Simultaneously, pancreatic stellate cells are activated either by cytokines during cannabis-induced necroinflammation or directly by cannabinoid metabolism and resulting oxidative stress. These activated stellate cells then increase the synthesis of extracellular matrix proteins, ultimately causing pancreatic fibrosis. This cascade of events describes the progression from cellular changes to the development of chronic pancreatitis in the context of cannabis abuse. Figure created in BioRender.com.

The pathophysiology of CIP is complex and multifaceted, involving the interplay between cannabinoid receptors, pancreatic cells, and inflammatory mediators (Figure 2). Understanding these mechanisms is crucial for elucidating the role of cannabis in pancreatic inflammation and developing targeted therapeutic approaches.

Cannabinoid receptors, primarily cannabinoid receptor type 1 and type 2 (CB1 and CB2), are widely distributed throughout the body, including the pancreas [14]. CB1 receptors are predominantly expressed in the exocrine pancreas, particularly in the acinar cells responsible for enzyme production. In contrast, CB2 receptors are more abundant in pancreatic islet cells [15]. The presence of these receptors suggests that the pancreas is a target organ for cannabinoids, both endogenous and exogenous.

Several mechanisms have been proposed to explain how cannabis use may lead to pancreatic inflammation. One hypothesis involves the direct activation of CB1 receptors in pancreatic acinar cells by exogenous cannabinoids. This activation may lead to increased intracellular calcium levels, triggering premature activation of digestive enzymes within the acinar cells [16]. The resulting autodigestion of pancreatic tissue is a hallmark of AP.

Another proposed mechanism involves the modulation of pancreatic secretion by cannabinoids. Studies have shown that CB1 receptor activation can inhibit cholecystokinin-induced amylase secretion from pancreatic acinar cells [17]. This alteration in normal pancreatic secretion patterns may lead to the accumulation of digestive enzymes within the pancreas, potentially contributing to inflammation and tissue damage.

Cannabis use may also affect pancreatic blood flow through its action on vascular cannabinoid receptors. Activation of these receptors can lead to vasoconstriction, potentially reducing pancreatic perfusion and creating an ischemic environment conducive to inflammation [18]. This effect may be particularly relevant in cases of heavy or chronic cannabis use.

The interaction between cannabis and other risk factors for pancreatitis is an area of growing interest. Alcohol consumption, a well-established cause of AP, may have synergistic effects with cannabis use. Both substances can independently activate CB1 receptors in the pancreas, potentially exacerbating their pro-inflammatory effects when used concurrently [19]. This interaction may explain the higher incidence of pancreatitis observed in individuals who use both cannabis and alcohol compared to those who use either substance alone [10].

Genetic factors may also influence an individual's susceptibility to CIP. Polymorphisms in genes encoding cannabinoid receptors or enzymes involved in endocannabinoid metabolism could potentially modulate the pancreatic response to exogenous cannabinoids [20]. For example, variations in the CNR1 gene, which encodes the CB1 receptor, have been associated with altered susceptibility to various inflammatory conditions and may play a role in CIP [21].

The role of the endocannabinoid system in pancreatic homeostasis adds another layer of complexity to the pathophysiology of CIP. Under normal conditions, endocannabinoids play a role in regulating pancreatic function and may have protective effects against inflammation [22]. However, chronic exposure to exogenous cannabinoids may disrupt this delicate balance, leading to dysregulation of the endocannabinoid system and increased susceptibility to pancreatic injury.

It is important to note that the effects of cannabis on the pancreas may vary depending on the specific cannabinoids involved. While Δ9-tetrahydrocannabinol (THC) is primarily responsible for the psychoactive effects of cannabis and may contribute to pro-inflammatory mechanisms, other cannabinoids like cannabidiol (CBD) have shown anti-inflammatory properties in some studies [23]. This dichotomy highlights the need for further research to elucidate the differential effects of various cannabis components on pancreatic health.

Clinical presentation

The clinical presentation of CIP often mirrors that of AP from other etiologies, making it challenging to distinguish based solely on symptoms. However, certain features may raise suspicion of cannabis involvement, particularly in younger patients without other apparent risk factors.

The cardinal symptom of cannabis-induced AP is severe epigastric pain, typically described as constant and radiating to the back [24]. This pain may be accompanied by nausea and vomiting, which can be severe and persistent. Patients often report anorexia and may develop tachycardia and hypotension in more severe cases. Physical examination may reveal epigastric tenderness, abdominal distension, and, in some cases, signs of systemic inflammation such as fever and tachypnea [5].

While most cases of CIP present as acute episodes, there is growing evidence to suggest that chronic cannabis use may lead to recurrent AP or even chronic pancreatitis in some individuals [25]. Chronic presentations may be characterized by persistent or intermittent abdominal pain, weight loss, and signs of pancreatic insufficiency such as steatorrhea and malabsorption. These cases often pose significant diagnostic challenges, as the relationship between ongoing cannabis use and pancreatic inflammation may be less apparent.

The diagnosis of CIP relies on a combination of clinical, laboratory, and imaging findings. Elevated serum lipase and amylase levels are typically observed, with lipase being more specific for pancreatic inflammation [3]. Imaging studies, such as contrast-enhanced computed tomography (CECT) or magnetic resonance imaging (MRI), may reveal pancreatic edema, peripancreatic fluid collections, or other features consistent with AP [26].

However, diagnosing cannabis as the specific etiology presents several challenges. First, many patients may be reluctant to disclose their cannabis use due to legal concerns or social stigma, necessitating a thorough and non-judgmental history-taking approach [27]. Second, the temporal relationship between cannabis use and the onset of pancreatic symptoms can vary, with some cases occurring after prolonged use and others following acute intoxication [5].

Furthermore, the lack of standardized diagnostic criteria specifically for CIP complicates its identification and classification. Currently, the diagnosis is often one of exclusion, made after ruling out other common causes of AP such as gallstones, alcohol, and certain medications [6]. This process may involve additional tests, including liver function tests, triglyceride levels, and in some cases, genetic testing for hereditary pancreatitis.

The potential for cannabis to exacerbate pancreatitis caused by other factors further complicates the diagnostic picture. In cases where multiple risk factors are present, determining the relative contribution of cannabis to pancreatic inflammation can be challenging [19]. This underscores the importance of considering cannabis use in the context of a patient's overall risk profile for pancreatitis.

Clinicians should maintain a high index of suspicion for CIP, particularly in younger patients presenting with idiopathic AP. A detailed history of substance use, including the frequency, duration, and method of cannabis consumption, is crucial for accurate diagnosis [8]. Additionally, urine toxicology screening for cannabinoids may be helpful, although it cannot distinguish between recent use and chronic exposure.

Diagnosis

The diagnosis of CIP requires a comprehensive approach that integrates clinical presentation, laboratory findings, imaging studies, and careful consideration of differential diagnoses. As with other forms of AP, early and accurate diagnosis is crucial for optimal management and prevention of complications.

Laboratory findings play a pivotal role in the initial assessment of suspected pancreatitis. Serum lipase is considered the most specific and sensitive marker for pancreatic inflammation, with levels typically elevated to at least three times the upper limit of normal in AP [3]. While amylase is also commonly measured, it is less specific and can be elevated in various non-pancreatic conditions. In CIP, these enzyme elevations may be particularly pronounced, with some studies reporting higher median lipase levels compared to other etiologies [5].

Additional laboratory tests are essential for evaluating the severity of pancreatitis and identifying potential complications. These include complete blood count, metabolic panel, liver function tests, and triglyceride levels. Elevated white blood cell count and C-reactive protein levels may indicate systemic inflammation, while abnormal liver enzymes could suggest biliary involvement or concomitant liver injury [28]. Serum calcium levels should be monitored, as hypocalcemia can be associated with severe pancreatitis.

Toxicology screening for cannabinoids in urine or blood can provide objective evidence of recent cannabis use. However, it's important to note that these tests cannot definitively establish cannabis as the cause of pancreatitis, as they do not indicate the timing or quantity of consumption [29]. Furthermore, the presence of synthetic cannabinoids may not be detected by routine screening tests, necessitating specialized assays in some cases.

Imaging studies are crucial for confirming the diagnosis of AP and assessing its severity. CECT is the gold standard imaging modality, offering detailed visualization of pancreatic and peripancreatic structures [21]. Typical findings in AP include pancreatic enlargement, peripancreatic fat stranding, and fluid collections. CECT can also help identify complications such as necrotizing pancreatitis or vascular involvement.

MRI provides an alternative to CT, particularly in cases where iodinated contrast is contraindicated or when a more detailed evaluation of the pancreatic and biliary ducts is needed [30]. MRI may be superior in detecting subtle parenchymal changes and characterizing fluid collections.

Transabdominal ultrasonography is often performed early in the diagnostic workup, primarily to evaluate for gallstones and biliary tract dilatation. While less sensitive for pancreatic pathology, ultrasound can be useful for follow-up imaging and monitoring of fluid collections [31].

The differential diagnosis of CIP is broad and includes other common causes of AP. Gallstone pancreatitis must be carefully excluded, as it represents the most frequent etiology in many populations. Alcohol-induced pancreatitis is another important consideration, particularly given the frequent co-occurrence of alcohol and cannabis use [19]. Other potential causes include hypertriglyceridemia, medication-induced pancreatitis, autoimmune pancreatitis, and pancreatic tumors.

Distinguishing CIP from these other etiologies often requires a thorough history, including detailed substance use patterns, and exclusion of alternative causes through appropriate testing. The presence of other risk factors does not necessarily rule out a contributory role of cannabis, as multiple factors may interact to precipitate pancreatic inflammation [8].

In cases of recurrent AP or suspected chronic pancreatitis, additional diagnostic modalities may be employed. Endoscopic ultrasound can provide high-resolution imaging of the pancreas and surrounding structures, while also allowing for fine-needle aspiration if a mass lesion is identified [32]. Genetic testing for mutations associated with hereditary pancreatitis may be considered in younger patients or those with a family history of pancreatic disease. Table 1 summarizes the different diagnostic approaches in CIP.

**Table 1:** Different diagnostic methods used in cannabis-induced pancreatitis

Study	Diagnostic Technology	Advantages	Disadvantages
Russo [21]	Contrast-enhanced computed tomography (CECT)	Gold standard imaging; detailed visualization of pancreatic and peripancreatic structures.	Exposure to radiation; potential contrast-related complications.
Tenner et al. [3]	Serum lipase test	Most specific and sensitive marker for pancreatic inflammation	-
Working Group IAP/APA Acute Pancreatitis Guidelines [28]	Complete blood count, metabolic panel, liver function tests	Evaluates severity of pancreatitis and identifies potential complications.	-
Heinz et al. [29]	Toxicology screening	Provides objective evidence of recent cannabis use.	Cannot establish cannabis as a definitive cause or indicate timing/quantity of consumption.
Zaheer et al. [30]	Magnetic resonance imaging (MRI)	Alternative to CT; better for detailed evaluation of pancreatic and biliary ducts; superior in detecting subtle parenchymal changes.	Longer scan time; higher cost; not suitable for patients with certain implants.
Karmazanovsky et al. [31]	Transabdominal ultrasonography	Useful for evaluating gallstones and biliary tract dilatation; good for follow-up imaging and monitoring fluid collections.	Less sensitive to pancreatic pathology.
Fusaroli et al. [32]	Endoscopic ultrasound	High-resolution imaging of the pancreas and surrounding structures; allows for fine-needle aspiration.	Invasive procedure; requires sedation.

Management and treatment

The management of CIP follows the general principles established for AP of other etiologies, with some specific considerations related to cannabis use. The primary goals of treatment are to provide supportive care, prevent complications, and address the underlying cause.

Acute management strategies focus on aggressive fluid resuscitation, pain control, and nutritional support. Intravenous fluid therapy is crucial in the early stages to maintain adequate intravascular volume and prevent hypovolemic shock [26]. Balanced crystalloid solutions are preferred, with the rate and volume guided by clinical parameters such as urine output, heart rate, and blood pressure. Pain management typically involves opioid analgesics, although there is growing interest in non-opioid alternatives to reduce the risk of addiction, particularly in patients with a history of substance use [33].

Nutritional support is an essential component of acute management. While traditional practice involves bowel rest, current evidence supports early enteral nutrition in most cases of AP [34]. Enteral feeding helps maintain gut barrier function and reduces the risk of infectious complications. In cases of severe pancreatitis or persistent intolerance to enteral feeding, parenteral nutrition may be necessary.

Monitoring for and managing complications is crucial in the acute phase. This includes regular assessment for signs of organ failure, pancreatic necrosis, and infected collections. In cases of biliary pancreatitis, early endoscopic retrograde cholangiopancreatography (ERCP) may be indicated if there is evidence of concurrent cholangitis or persistent biliary obstruction [35].

Long-term treatment approaches for CIP focus on preventing recurrence and managing any chronic sequelae. The cornerstone of long-term management is cannabis cessation. Patients should be counseled on the importance of abstaining from cannabis use to reduce the risk of recurrent pancreatitis [19]. This often requires a multidisciplinary approach involving addiction specialists, psychologists, and support groups.

For patients who develop chronic pancreatitis as a result of recurrent cannabis-induced acute episodes, management strategies may include enzyme replacement therapy for exocrine insufficiency, pain management protocols, and nutritional counseling [36]. Regular follow-up with a gastroenterologist or pancreatologist is essential to monitor for progression of pancreatic disease and address any emerging complications.

Cannabis cessation has a significant impact on outcomes in patients with CIP. Studies have shown that continued cannabis use is associated with an increased risk of recurrent attacks and progression to chronic pancreatitis [8]. Conversely, abstinence from cannabis has been linked to a reduced frequency of acute episodes and improved long-term pancreatic function [5].

Implementing effective cannabis cessation strategies can be challenging due to the potential for dependence and withdrawal symptoms. A stepped approach to cessation may be necessary, involving cognitive-behavioral therapy, motivational enhancement, and in some cases, pharmacological interventions to manage withdrawal symptoms [37]. It is important to address any underlying mental health issues that may contribute to cannabis use, as these can impact the success of cessation efforts.

Patient education plays a crucial role in both acute and long-term management. Individuals should be informed about the potential risks of cannabis use on pancreatic health and the importance of disclosing their cannabis use to healthcare providers [24]. Education should also cover strategies for relapse prevention and the recognition of early signs of recurrent pancreatitis.

In cases where complete cannabis cessation is challenging, harm-reduction strategies may be considered. These might include counseling on safer consumption methods, avoiding high-potency products, and reducing the frequency of use [38]. However, it should be emphasized that the goal remains complete abstinence to minimize the risk of recurrent pancreatitis.

As research in this field progresses, there is a need for more targeted treatment approaches specific to CIP. This may include investigating the potential role of cannabinoid receptor antagonists in managing acute episodes or developing screening protocols to identify individuals at higher risk of cannabis-related pancreatic complications [22]. Ongoing clinical studies and long-term follow-up of affected individuals will be crucial in refining management strategies and improving outcomes for patients with CIP.

Prognosis and complications

The prognosis of CIP is generally favorable when compared to other etiologies of AP, particularly in cases where cannabis use is discontinued. However, the potential for recurrence and progression to chronic pancreatitis underscores the importance of early recognition and intervention. Short-term outcomes for CIP are typically good, with most patients experiencing resolution of symptoms within a few days of hospitalization and supportive care. The mortality rate for CIP is lower than that of AP from other causes, likely due to the younger age and fewer comorbidities of affected individuals. However, severe cases can still occur, with the potential for complications such as pancreatic necrosis, organ failure, and systemic inflammatory response syndrome [39].

Long-term outcomes largely depend on whether patients abstain from cannabis use following their initial episode. Those who achieve and maintain abstinence have a significantly reduced risk of recurrence and progression to chronic pancreatitis. However, continued cannabis use is associated with poorer long-term outcomes, including an increased likelihood of developing chronic pancreatitis and its associated complications [40]. Recurrence rates for CIP vary widely in the literature, ranging from 16% to 41% in different studies. This variability likely reflects differences in follow-up duration, cannabis cessation rates, and the presence of other risk factors. Factors associated with higher recurrence rates include continued cannabis use, higher frequency and duration of use prior to the initial episode, concurrent use of alcohol or other substances, and the presence of genetic predisposition to pancreatitis [5,41].

The potential complications of CIP can be divided into local and systemic categories. Local complications include pancreatic pseudocysts, which are fluid collections that may develop in and around the pancreas; pancreatic necrosis, which involves the death of pancreatic tissue and can lead to infection; and pancreatic duct disruption, which can result in damage to the pancreatic duct system, potentially leading to pancreatic fistulas [42]. Systemic complications, while less common in CIP compared to other forms of AP, can still occur and may include acute respiratory distress syndrome (ARDS), acute kidney injury, cardiovascular complications such as myocardial depression, and disseminated intravascular coagulation (DIC) [43].

A particularly concerning long-term complication is the development of chronic pancreatitis, characterized by irreversible structural changes to the pancreas and persistent inflammation. This can lead to chronic pain, exocrine and endocrine pancreatic insufficiency, and an increased risk of pancreatic cancer. The risk of progression to chronic pancreatitis is significantly higher in individuals who continue to use cannabis after their initial acute episode [44,45].

Thus, while the immediate prognosis of CIP is generally favorable, the potential for recurrence and long-term complications highlights the critical importance of cannabis cessation. Healthcare providers should emphasize the significance of abstinence in preventing future episodes and maintaining pancreatic health. Long-term follow-up and multidisciplinary care are essential to monitor for and address any emerging complications, particularly in patients with recurrent episodes or those who struggle with cannabis cessation [46]. Table 2 provides an overview of the prognosis and complications associated with CIP, comparing short-term and long-term aspects.

**Table 2:** An overview of the prognosis and complications associated with cannabis-induced acute pancreatitis, comparing short-term and long-term aspects. ARDS: acute respiratory distress syndrome; DIC: disseminated intravascular coagulation

Aspect	Short-Term	Long-Term
General Prognosis [38]	Favorable compared to other etiologies	Depends on cannabis abstinence
Mortality Rate [39]	Lower than other causes of acute pancreatitis	Not specifically mentioned
Symptom Resolution [40]	Within a few days of hospitalization	Varies based on cannabis use
Recurrence Rate [5]	Not applicable	16% to 41%
Factors Affecting Outcomes [41]	Age, comorbidities	Cannabis cessation, frequency, and duration of prior use, concurrent substance use, genetic predisposition
Local Complications [42]	Pancreatic necrosis	Pancreatic pseudocysts, pancreatic necrosis, pancreatic duct disruption
Systemic Complications [43]	Organ failure, systemic inflammatory response syndrome	ARDS, acute kidney injury, cardiovascular complications, DIC
Risk of Chronic Pancreatitis [44,45]	Not applicable	Higher with continued cannabis use
Key Recommendations [46]	Early recognition and intervention	Cannabis cessation, long-term follow-up, multidisciplinary care

Special considerations

Cannabis use in patients with pre-existing pancreatic conditions presents a complex clinical scenario that requires careful consideration. For individuals with chronic pancreatitis or a history of AP, cannabis use may exacerbate their condition or trigger recurrent episodes [40]. The interaction between cannabis and compromised pancreatic function is not fully understood, but it is hypothesized that cannabinoids may further disrupt already altered pancreatic secretion patterns or exacerbate underlying inflammation [22]. Consequently, healthcare providers should counsel patients with pre-existing pancreatic conditions about the potential risks associated with cannabis use and strongly advise abstinence to prevent complications.

The use of medical cannabis and its impact on pancreatitis risk is an area of ongoing debate and research. While some studies have suggested the potential anti-inflammatory properties of certain cannabinoids, particularly CBD, the overall effect of medical cannabis on pancreatic health remains unclear [23]. Patients prescribed medical cannabis for other conditions should be monitored closely for signs of pancreatic inflammation, especially if they have other risk factors for pancreatitis. Furthermore, the method of consumption (e.g., smoking versus oral ingestion) may influence the risk profile, with smoking potentially carrying a higher risk due to the additional exposure to combustion products [11].

Several significant gaps exist in our current knowledge of CIP. The exact mechanisms by which cannabis triggers or exacerbates pancreatic inflammation are not fully elucidated. Additionally, the dose-response relationship between cannabis use and pancreatitis risk is poorly defined, making it challenging to establish clear guidelines for safe consumption levels, if any. The long-term consequences of chronic cannabis use on pancreatic function, particularly in young adults, remain inadequately studied. Moreover, the potential interaction between cannabis and other pancreatitis risk factors, such as alcohol consumption or genetic predisposition, requires further investigation [19].

These knowledge gaps highlight several potential areas for further investigation. Prospective cohort studies are needed to better characterize the incidence and natural history of CIP, particularly in regions where cannabis use is legal and more prevalent. Such studies could help identify specific risk factors and establish more precise diagnostic criteria for CIP. Basic science research focusing on the molecular mechanisms of cannabinoid action in pancreatic tissue could provide insights into the pathophysiology of CIP and potentially identify novel therapeutic targets.

Another crucial area for investigation is the development of standardized screening tools for cannabis use in patients presenting with AP. These tools could help clinicians more accurately identify cannabis as a potential etiologic factor and guide appropriate management strategies. Additionally, research into the potential protective or therapeutic effects of specific cannabinoids, such as CBD, in pancreatic inflammation could open new avenues for treatment.

Lastly, given the increasing prevalence of cannabis use worldwide, public health research is needed to assess the impact of various cannabis policies on the incidence of pancreatitis and to develop effective prevention strategies. This could include studies on the effectiveness of educational interventions about the risks of cannabis use on pancreatic health and evaluations of harm reduction approaches for individuals unable to achieve complete abstinence.

## Conclusions

CIP is an emerging and potentially underrecognized cause of pancreatic disease, particularly among younger populations. This review has explored the connection between cannabis use and pancreatic inflammation, including its epidemiology, pathophysiology, clinical presentation, and management. While the short-term prognosis is often favorable, the risk of recurrence and chronic pancreatitis highlights the importance of cannabis cessation. With increasing global cannabis use, healthcare providers must be vigilant, especially in idiopathic cases. Addressing knowledge gaps through targeted research and a multidisciplinary approach is crucial to improving outcomes and understanding the impact of cannabis on pancreatic health.
